# Mene Mene Tekel Upharsin: Clerical Speed and Elementary Cognitive Speed are Different by Virtue of Test Mode Only

**DOI:** 10.3390/jintelligence7030016

**Published:** 2019-07-09

**Authors:** Florian Schmitz, Oliver Wilhelm

**Affiliations:** Institute of Psychology, Ulm University, Albert-Einstein Allee 47, 89081 Ulm, Germany

**Keywords:** mental speed, assessment mode, paper and pencil, computer-based assessment

## Abstract

Current taxonomies of intelligence comprise two factors of mental speed, clerical speed (Gs), and elementary cognitive speed (Gt). Both originated from different research traditions and are conceptualized as dissociable constructs in current taxonomies. However, previous research suggests that tasks of one category can be transferred into the other category by simply changing the mode of administration, i.e., in form of a paper-and-pencil test or in from of a computer-based elementary cognitive task. However, cross-mode correlations for specific tasks are usually only moderate. In the present study, mental speed was assessed as a broad construct across different tasks and stimulus materials. This allowed modeling mental speed as a hierarchical construct for paper-and-pencil as well as for computer-based tests. Cross-mode correlations of the respective general factors were moderate (*r* = 0.64), while the cross-mode correlations of task-specific components depended on task type (*r* = 0.12 to *r* = 0.71). Only the g factors of mental speed, but not the task-specific components, were found to be related with working memory capacity as a marker of cognitive ability. The speed general factor modeled across computer-based tests was more highly correlated with working memory capacity (*r* = 0.66) than the general factor modeled across paper-and-pencil tests (*r* = 0.46). These findings corroborate a crucial role of the assessment method and imply that validity of speed tests is affected by the choice of the test format.

## 1. Introduction

“Mene mene tekel upharsin” is known as the “writing on the wall” mentioned in the Bible (Daniel 5:25) and has become a metaphoric warning of misfortune. Its translation is “numbered, numbered, weighed (and found wanting), divided,” and could be interpreted as assessed and found to be insufficient in the present context. We suggest that the still widely held distinction between clerical speed and elementary cognitive speed is flawed and that unwanted issues of administration mode are responsible for the apparent dissociation of both speed factors.

Mental speed refers to the ability to solve simple task efficiently in limited time. Consequently, tests that tap mental speed comprise intellectually simple tasks [1] that are easy enough that all participants could reach one hundred percent accuracy given sufficient time [2]. The relevance of mental speed is reflected by the fact that all current models of intelligence comprise broad speed factors [3], including the Gf-Gc theory [4], the Three Stratum Theory [5], and the Cattell-Horn-Carrol (CHC) theory of cognitive abilities [6].

Additionally, to some lower-level specific speed factors, hierarchical models distinguish between two theoretically meaningful speed factors on the level of primary abilities. With respect to their origin and research traditions, they can be referred to as clerical (Gs) and elementary cognitive speed (Gt) [7]. In Carroll’s Three Stratum Theory [5], they are referred to as cognitive speediness (Gs) and response time (RT) and decision speed (Gt), whereas in McGrew’s CHC [6] theory, they are called processing speed (Gs) and decision/ reaction time speed (Gt), respectively.

### 1.1. Clerical Speed (Gs)

Originally, speed tasks were introduced into applied assessment to predict efficacy in clerical jobs [8,9]. Typical tasks require, for instance, searching and comparing numbers and letters. Completion of such basic tasks once was deemed important for office and administrational duties [8,9]. Today, speed tests are no longer confined to the pragmatic role of predicting efficacy in office work. Conversely, “clerical speed” now has the status of a broad ability factor in hierarchical models of intelligence [5]. As such, proponents of the psychometric view conceive mental speed as one important primary ability among others [10]. We will refer to this descriptive speed factor as “clerical speed” [7] in the following to emphasize its origin in applied assessment and its status in current psychometric taxonomies of intelligence.

Tests subsumed under the clerical speed factor are characterized by their simplicity. It is assumed that only few mental operations are required to complete them [5,10]. However, as their name suggests, speed tests are administered under high time pressure. Early definitions of mental speed emphasized as well the smoothness of response production, such as writing the answer [11]. Later, it was criticized [5] that it remains largely unresolved to what extent clerical speed is determined by peripheral factors such as screening the stimuli and motor execution as contrasted with more central processing requirements. Conventionally, clerical speed tests are administered in a paper-and-pencil (PP) mode. All items are printed on one page and participants are asked to complete as many of them as they can in the given time. The test score usually reflects the number of correctly solved items per time unit. In this respect, clerical speed tests are comparable with other ability tests that possess an accuracy-based performance metric.

### 1.2. Elementary Cognitive Speed (Gt)

There is a long lasting tradition of research addressing the relation of RT in simple reaction time tasks on the one side and intelligence on the other side, e.g., [12,13]. In this line of research, mental speed is assessed with so-called elementary cognitive tasks (ECTs) that purport to measure most basic cognitive operations. ECTs are frequently administered using special apparatuses or the computer and mental speed is measured as the time required per correct response. In part, this research was motivated by the assumption that elementary cognitive speed is the most fundamental mechanism underlying intelligence [14]. Thereby, proponents of the elementary cognitive speed view suggest an explanatory account of intelligence. Specifically, it was postulated that speed of encoding, speed of access to short-term memory, and speed of retrieval from long-term memory affects performance in cognitively more demanding tasks [15]. In fact, Jensen [16] proposed that speed in these elementary cognitive processes contributes to the g factor in conventional psychometric intelligence tests, i.e., the positive manifold observed across diverse ability tests.

One line of research supporting a link between elementary cognitive speed and intelligence comes from developmental psychology. Classical research supports a general age-related decrease in speed of information processing across adulthood to old age [17,18] that seems to affect all domains of mental speed: psychometric, experimental, and psychophysical [19]. However, there are also marked individual differences in the developmental trajectories [20] and individual differences in age-related changes in mental speed are clearly related with according changes in intelligence [21].

Further, cross-sectional data supports a moderate relation between elementary cognitive speed and intelligence (−0.22 ≤ *r* ≤ −0.45; see [22] for a meta-analysis), where fluid intelligence (Gf) was shown to be the primary ability consistently or most strongly related with elementary cognitive speed [10,23]. Additionally, a number of moderators of the speed-intelligence relation were identified [22], particularly the complexity of the speed tasks [18]. Another recent meta-analysis focusing on Hick-type speed tasks [24] confirmed the existence of a moderate relation between speed and intelligence in the magnitude of *r* = −0.18 for a simple 0-bit condition in the Hick task and *r* = −0.28 for a more complex 2-bit condition.

Additionally, to the only moderate relation observed for speed with intelligence, the well-replicated moderation through complexity challenges the view that basic speed alone is the crucial factor constituting positive manifold. Nevertheless, current accounts of positive manifold conceptualize speed of processing as one important factor, additionally to attentional resources and working memory capacity [25,26]. Following this notion, positive manifold is conceptualized as an emergent variable driven by the overlap of domain-general low-level mechanisms [27,28]. 

A number of special devices and apparatuses were invented to measure response times, including the early response time apparatuses in Galton’s anthropometric laboratory and Jensen’s response box. Today, most researchers use computer-based (CB) measures to administer stimuli and to collect responses [29,30]. Usually, individual stimuli are presented successively on screen and are classified by pressing buttons on the keyboard. Mean correct response time is the most frequently reported score, which is sometimes further subdivided into decision time and movement time. However, more sophisticated ways of performance modeling are possible, given that RT and accuracy information is available for each item (see [31] for a theoretical overview of RT modeling techniques), but these have been exploited only in a limited number of studies to date (e.g., [32,33,34,35]).

### 1.3. Separability of Speed Factors and Cross-Mode Transfer

Clerical speed (Gs) and elementary cognitive speed (Gt) originated from different field and are used in different research traditions. Further, different test materials are used, performance is scored differently, and both factors are clearly distinguished in current taxonomies of cognitive abilities [5,6]. In spite of this commonly held distinction, both speed constructs could be argued to be highly similar, as both are assessed with cognitively undemanding tasks that require quick responding. However, it was argued that “psychometric speed […] is something entirely different from the speed of information processing measured by RT [response time] or IT [inspection time]” ([36], p. 224). In part, this contention may be grounded in extensive psychometric research into the structure of human abilities that revealed two separable factors, e.g., [5], as well as the observation that both task classes possess differential validity for intelligence (i.e., somewhat higher relations for computerized ECTs; [37]).

Some findings suggest that it is not the nature of the respective task classes per se, but the mode of administration that is responsible for their dissociation. For instance, in one study [38], typical elementary cognitive tasks such as Coding, Sternberg, and Posner tasks were administered in a PP mode along with a battery of conventional PP tests of psychometric abilities. Results indicated that a general factor of mental speed could be modeled across all ECTs. In turn, this general factor of PP administered Gt tasks was found to be substantially related (r = 0.86) with a Gs factor that captures the communality of clerical speed tasks from the PP administered ability battery.

In another study [39] a considerably broader spectrum of ECTs were administered in PP mode, including conventionally PC administered tasks such as Task Switching and Substitution paradigms, but also tasks that are conventionally administered with special RT apparatuses, including Hick and Odd-Man-Out (OMO) paradigms. Results indicated that these task classes loaded on a common higher order factor of mental speed that was found to be perfectly related with a Gs factor modeled across conventionally PP administered Gs tests. Thereby, identity of an elementary cognitive speed (Gt) with a clerical speed (Gs) factor was demonstrated when tasks were administered in the PP mode. 

In an analogous study [40], typical paper-and-pencil speed task such as Search and Comparison tasks were administered in a computerized mode. A common factor of metal speed could be modeled across CB administered Gs tasks. In turn, this common factor of CB administered Gs tasks could be constrained to identity with a higher order factor of Gt tasks that were conventionally administered in CB mode. Thereby, identity of a clerical speed (Gs) factor and an elementary cognitive speed (Gt) factor could be demonstrated when all tasks were administered in CB mode. These findings imply that tasks conventionally assumed as tapping either clerical speed or elementary cognitive speed, respectively, are not that different at all. By changing their mode of administration, they can be transferred into the respective other class. This suggests a major role of the assessment mode for factorial validity of the speed tasks as well as for the dissociation of the derived factors.

### 1.4. Aims of This Study

The present study was conducted to test if the assessment mode is responsible for the apparent dissociation of speed factors. In line with current conceptualizations of mental speed as a hierarchical construct [10,39,40,41], we modeled mental speed as a broad factor across a battery of different clerical speed tests using different stimulus materials. This allowed investigating cross-mode equivalence concurrently at different levels of specificity, namely at the level of a general speed factor, and additionally at the level of task-specific components. Although we intended to model mental speed as a broad latent factor within each assessment mode, we predicted that the assessment mode would lead to the dissociation of both speed factors, as indicated by a cross-mode correlations considerably below unity.

Further, we aimed to test how assessment mode affected the speed-ability relation that is predicted by reductionist models in the tradition of elementary cognitive speed theory. In line with previous research [22,24], we predicted moderate relations between both g factors of mental speed with cognitive ability. The relations with cognitive ability were expected to be roughly comparable in magnitude for the speed factors derived from PP and CB speed tests (but see [37], for somewhat larger relations of CB tasks). Of course, moderate relations would be expected also from a psychometric-structural perspective, reflecting the positive manifold of different ability constructs. However, it is unresolved to what extent construct validity is affected by the assessment mode.

## 2. Materials and Methods

### 2.1. Sample

In this study, *n* = 129 participants participated. As paper-and-pencil tests from four participants were lost, they were excluded from the analyses. The sample comprised mostly of university students (*n* = 117) with an age of 22.0 years (*SD* = 3.1; range: 18–38); *n* = 105 indicated to be female and *n* = 19 to be male. Participants signed informed consent prior to participation and were debriefed after completing the study. They were compensated with 15 Euros or partial course credit. The work described in this study was carried out in accordance with the Code of Ethics of the World Medical Association (Declaration of Helsinki) for experiments involving humans. CB data from this project were analyzed in another study [42], but cross-mode equivalence has not been investigated.

### 2.2. Materials

#### 2.2.1. Paper-and-Pencil Speed Tests

In order to measure mental speed in a broad way, three types of speed tasks were employed that are typically used to assess the clerical speed factor, namely Search, Comparison, and Substitution tasks [10,30]. Construction of the speed tests followed a matrix design were each type of task was combined with three different materials: numbers, letters, and symbols. Each task from the test booklet was preceded by a separate instruction page with item examples. Participants were instructed to respond as quickly as they could without committing errors. A sufficient number of stimuli were presented in each task to avoid ceiling effects and participants were informed that people usually cannot complete all items in the given time. Upon a “start” command, participants turned the page and had 45 s to work on the actual test that was also formatted on one page. After the “stop” command, participants turned the page to the next instruction page. Prior to the relevant tests, participants completed a Connect-the-Numbers test as an ice-breaker task. In this test, they had to connect 20 numbers that were randomly distributed across the page in increasing order.

In Search tasks, stimuli were presented in rows and target stimuli had to be canceled. In the task version with numbers, single digits from 0 to 9 were displayed in Calibri font (15 pt.). The rows were separated by thin horizontal lines to help participants focus their attention and to make sure that they would complete the task line by line, which was relevant for scoring the task. Participants had to cancel all numbers ‘3’ as target stimuli. In total, 450 stimuli were shown on the test page, in 18 rows with 25 numbers each. In the task with letters, both consonants and vowels were presented as stimuli, and participants had to cancel all letters ‘A’. Again, 450 stimuli were shown on the page, presented in 18 rows with 25 stimuli each. In the task version with symbols, three simple emoticons were chosen as stimuli from the Wingdings font (18 pt.). They looked very similar and could be distinguished only by the curvature of the line representing their mouth. The smiling emoticon served as the target. A total of 360 stimuli were shown on the page, displayed in 18 rows with 20 stimuli each. 

In Comparison tasks, participants were shown two triples of stimuli presented horizontally aligned. Their task was to mark if the triples were identical or not by writing an “=” or “×” in the middle between the two triples, respectively. In case of a mismatch, only one of the three stimuli in one pair was replaced by a different stimulus. The pairs of triples were presented in three columns on the test page, separated by thin vertical lines on each side to help participants focus their attention and to walk through the pairs item by item, and column by column. In the task version with numbers, digits from 0 to 9 were presented in Calibri font (16 pt.). A total of 75 pairs were presented, 25 in each of the three columns. In the task with letters as stimuli, only consonants were combined to avoid readable syllables. Additionally, it was checked that the triples did not form familiar acronyms. A total of 60 pairs were presented, 20 in each of the three columns. In the task version with symbols, 10 simple symbols from the Wingdings 2 font (16 pt.) were selected as stimuli. There were 60 pairs in total, 20 in each of the three columns. 

In the Substitution tasks, participants were given a coding table showing how 9 stimuli of a first category (e.g., digits) had to be substituted by 9 stimuli of a second category (e.g., symbols). Participants were asked to familiarize themselves with the coding table on the instruction page, additionally the coding table was also printed on the top of the test page so that participants could look up the substitution rules occasionally. The stimuli were presented in grids with two rows: The stimuli of the first category were presented in the upper cells of the grid, while participants had to draw the corresponding stimuli of the second category into the lower cells. They were instructed to work through the task line by line from the left to right. The first task version required the substitution of numbers by symbols. Numbers were drawn from the digits from 1 to 9 and presented in Calibri font (20 pt.). A total of 50 stimuli of the first category were shown, presented in 5 rows (grids) with 10 stimuli each. In the second task version, symbols had to be substituted by letters. Stimuli of the first category were simple symbols drawn from the Wingdings 2 font (18 pt.), and all stimuli required for the second category were consonants. Again, 50 stimuli of the first category were displayed in 5 rows with 10 stimuli each. In the third version of this task, letters had to be substituted by numbers. The stimuli of the first category were all consonants presented in Calibri font (20 pt.). Of them, 50 were presented in 5 rows with 10 stimuli each.

**Figure 1:**
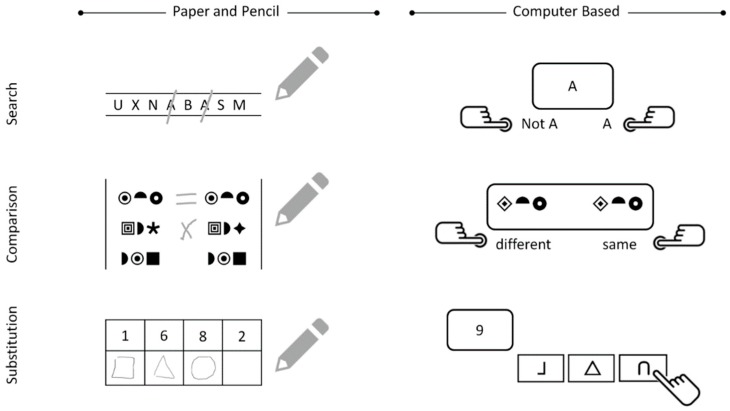
Examples of paper-and-pencil and computer-based mental speed tests.

Examples of the speed tasks are displayed in Figure 1. As typically done, we computed scores that reflected the number of correct responses in the given time. In Search tasks, the last stimulus marked by the participant was interpreted as indexing the maximum number of processed stimuli. False alarms and omissions were subtracted from that number to yield the number of correctly solved items in the given time. In Comparison and Substitution tasks, the number of responses directly corresponds with the number of processed stimuli. Accordingly, the scores directly reflected the sum of correctly solved items in the given time.

#### 2.2.2. Computer-Based Speed Test

Construction of the computerized speed tests closely followed the paper-and-pencil tests. However, we decided to administer tasks in a most typical way for their respective modes of presentation, see [29]. For the CB tasks, this meant presenting stimuli individually on a trial-wise basis on screen and using a customized computer keyboard for response collection—constituting a prototypical setting for CB testing. Stimuli in the computerized tasks were drawn from the same stimulus sets as in the PP tasks. Stimuli were presented in pseudo-random order in the CB tasks, hence identical for all participants. All stimuli were presented in black font on a light gray screen (with red, green, blue (RGB) additive color components = 220, 220, 220) of a 22’ thin-film transistor monitor. Customized computer keyboards were used for data collection, which allowed replacing the labels of response keys, e.g., with symbols. After a response was elicited, the screen was cleared, and the next stimulus was presented after 500 ms in all CB tasks. No error feedback was provided (as in PP tests). The tasks were controlled by a compiled C++ program using the Simple DirectMedia Layer libraries for stimulus presentation and response collection.

In the Search tasks, stimuli were successively presented in the center of the monitor. Participants classified each stimulus as target or non-target by pressing a right or left response key, respectively. Identical stimuli served as targets in the three versions of the PP tasks. Numbers and letter were presented in LucidaSansRegular font (60 pt.) and smileys were presented as pictures in a comparable size. Participants completed two blocks of each task, comprising a total of 135 trials of the task with numbers, and 120 trials of the tasks with letters and symbols (plus two warm-up trials at the beginning of each block that were not analyzed).

In each trial of the Comparison tasks, a pair of two triples was presented horizontally aligned in the middle of the screen. Participants classified the two triples as identical or different by pressing a right or left response key, respectively. Numbers and letters were presented in LucidaSansRegular font (50 pt.), and symbols as pictorial stimuli in a comparable size. Participants completed two blocks of each task version, comprising 60 trials each (plus two warm-up trials). 

In the Substitution tasks, stimuli of the first category were presented successively in the center of the screen in LucidaSansRegular font (50 pt.). The coding table showing how stimuli of the first category were assigned to stimuli of the second category was visible in the lower part of the screen throughout the entire block. Customized keyboards were used for response collection: There was a “home” button in the middle of the keyboard and nine response keys were aligned behind the home button displaying the stimuli of the second category. Keyboards were exchanged between tasks so that response keys always showed the currently relevant stimuli of the second category. Participants were instructed to keep the home button suppressed with the index finger of their dominant hand. After having classified the presented stimulus, they responded by pressing the according response key. Participants completed two blocks of 60 trials (plus two warm-up trials) of each task. 

In all CB tasks, warm-up trials and extreme values according to the liberal Tukey [43] criterion (i.e., slower than 3 interquartile ranges on top of the 75 percentile of the RT distribution or below 200 ms) were removed from the analyses (across all speed variables *M* = 1.5%, *SD* = 1.1%, range: 0.2–3.6%). Tasks were scored by computing the mean rate of correct responses per time unit (1/RT). To this end, the raw RT values of correct responses were inserted as denominators into a fraction (1 s/RT), then, all fractions were averaged. Hence, this score denotes the number of responses per second for correct trials. It was chosen as it corresponds with conventional scoring in PP tests and because it normalizes the distribution of scores as an additional benefit for the correlational analyses.

#### 2.2.3. Working Memory Capacity (WMC) Tasks

We chose WMC as a criterion variable for cognitive functioning, as working memory is well defined from a theoretical perspective. Additionally, it is conceived a key aspect of decontextualized, mechanical, and fluid intelligence. In line with this contention, it was demonstrated to be substantially correlated with tests of intelligence in previous research [44,45,46,47]. We decided to assess WMC with Recall-1-Back tasks [48], which are recall-versions of the classical N-Back task that require retrieval of a stimulus that was presented between one and four trials ago. Three indicator tasks with different materials (numbers, letters, and symbols) were included in the battery. Thereby, all task classes (PP and CB speed as well as WMC) were administered with the same types of stimuli. This seemed to be indicated as some research suggests that stimulus material can affect relations of WMC tasks [49]. 

In the task version with numbers and letters, two to four rectangles were presented horizontally aligned, depending on the run. Each run started by showing stimuli in all rectangles. Then, only one stimulus appeared unpredictably in one of the rectangles in each trial. Participants had to type in the last stimulus previously shown in this rectangle. Depending on trial type, this could require recalling a stimulus in the directly preceding trial (n-1) to recalling a stimulus that was shown up-to four trails ago (n-4). Hence, retrieval requirements varied unpredictably across trials, constituting different levels of working memory load.

Numbers and letters were shown in LucidaSansRegular font (40 pt.) and the rectangles had a size of 150 pt. × 150 pt. The task version with symbols followed the same logic. However, symbols were presented in a 3 × 3 grid and participants had to indicate by mouse click the cell where the currently shown stimulus appeared the last time. Stimuli were drawn from nine simple symbols (size 79 pt. × 79 pt.) and presented in the cells (size 150 pt. × 150 pt.) of the grid. In all task versions, stimuli were shown for 3000 ms, which also corresponded with the trial response deadline. After that, stimuli were removed, but rectangles or grid, respectively, remained visible on screen. Task requirements varied from run to run in terms of working memory loads (1, 2, 3, or 4 stimuli to remember) and updating (6, 9, or 12 updates). Participants completed 3 runs (with a total 21 individual responses) in a practice phase and 12 runs (108 responses) in a test phase. Partial credit scoring [50] was applied, i.e., the proportion of correct responses from the test phase was computed in each WMC task.

### 2.3. Procedure

Data were collected in form of group testing with up to six persons at a time. The session started with the PP tests of clerical-perceptual speed that were administered in the order in which they were described above. The test managers gave standardized instructions and provided additional explanation if required. Test time for each test was 45 s, and the total time for the PP speed battery did not exceed 10 min. Then, participants took place in front of computers and launched the PC tests. Instructions were presented on screen, however, the test manager remained in the room to provide further explanation if required. Additionally, to the tests described above, two rapid serial visual presentation (RSVP) tasks were employed (results are reported in [42]). The computerized session started with one of the RSVP tasks. Next, the PC tests of elementary cognitive speed were presented in the order as described above. Then, the second RSVP task was administered and finally the three WMC tasks with numbers, symbols, and letters. Two short pauses were included in the task battery, one prior to the PC tasks and another one (of at least 5 min) prior to the WMC tasks. Additionally, restarting each task block was self-paced, so that participants could make more short pauses if they wished. The entire battery took about 1.5 h to complete. 

### 2.4. Data Analyses

**Figure 2:**
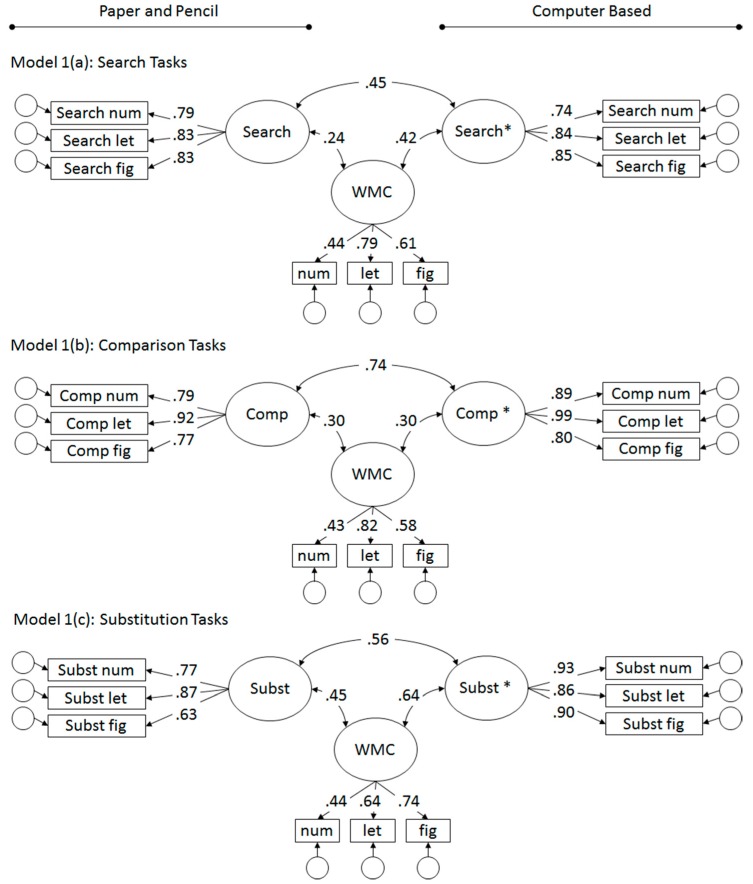
Relations of single task paper-and-pencil (PP) and CB speed factors across mode of administration, and with Working Memory Capacity (WMC), separate for 1(a) Search, 1(b) Comparison, and 1(c) Substitution tasks.

The analyses were conducted with R [51]. The psych package [52] was used for psychometric analyses, lavaan [53], and semTools [54] for structural equation modeling. First, equivalence of the PP and CB tests was tested separately for the three task classes (Search, Comparison, and Substitution tasks) by testing relations of latent factors for PP, CB, and WMC tasks (see Figure 2, Models 1(a–c)).

**Figure 3:**
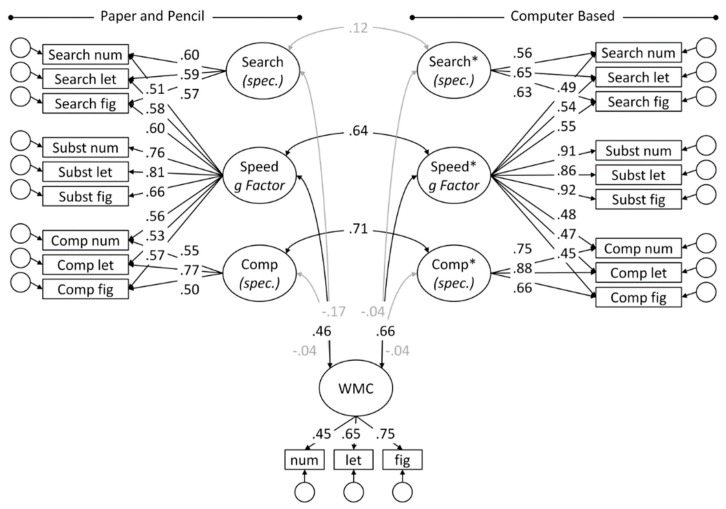
Relations of bifactor models fitted to PP and CB speed task across mode of administration, and with WMC, jointly for Search, Comparison, and Substitution tasks (Model 2).

Next, bifactor models were fitted across all speed tasks, separately for PP and CB tasks, with Substitution as the reference method (CTC(M-1); [55]), and nested, uncorrelated factors for the method specificity of Search and Comparison tasks (see Figure 3, Model 2). Previous research with the employed speed tasks, e.g., [33], has shown that this is the most parsimonious model with respect to structure and relation with WMC. The hierarchical models allowed testing equivalence of PP and CB administered tasks simultaneously for the broad g factors of mental speed as well as for task specific requirements. Models were evaluated taking into account several fit indexes, e.g., [56,57]: the chi-squared statistic (χ²), the root mean square error of approximation (RMSEA) with its 95% confidence interval, the standardized root mean square residual (SRMR), and the comparative fit index (CFI). Rules of thumb for an acceptable model fit include an RMSEA < 0.06, an SRMR < 0.08, and a CFI > 0.95 [56], although it was argued that these criteria may be too strict for small samples (*n* < 500) [58]. Nested models were compared with χ² difference tests. Additionally, the model comparison indexes Akaike information criterion (AIC) and Bayesian information criterion (BIC) were computed, both of which simultaneously take into account model fit and parsimony. These indexes favor the model with a lower value.

## 3. Results

### 3.1. Preliminary and Separate Analyses for Task Classes

Descriptive statistics for the paper-and-pencil as well as for the computer-based speed scores are given in Table 1. For all task classes, the stimulus-specific tasks loaded substantially on their respective factors and the (unconstrained) models displayed decent fit, with all RMSEA ≤ 0.07, all CFI ≥ 0.97 (see Appendix A for additional fit indices). For all task classes, cross-mode correlations were of moderate magnitude, ranging from *r* = 0.45 for the Search tasks to *r* = 0.74 for the Comparison tasks (see Figure 2).

The relations across test mode were tested for significance by fixing the correlation between latent variables to either zero or unity. Model fit as well as difference tests for the constrained, nested models relative to their respective unconstrained parent model are provided in Appendix A. None of the cross-mode correlation between latent factors could be constrained to zero or one. This confirmed the moderate cross-mode relations, corresponding with 20 to 55% shared variance in the current study. The rest of the factor variance corresponded with reliable task-specificity within PP and CB mode of administration.

The speed factors revealed moderate relations with WMC, although of variable strength in the range of *r* = 0.24 (for PP Search tasks) to *r* = 0.64 (for CB Substitution tasks). Relations with WMC were descriptively higher (by about 20 points) for the CB based speed factors when compared with PP based speed factors for the Search (*r* = 0.42, CI: 0.22–0.61; *r* = 0.24, CI: 0.02–0.46) and for the Substitution tasks (*r* = 0.64, CI: 0.49–0.80; *r* = 0.45, CI: 0.25–0.65). Differently for the Comparison tasks, virtually identical relations were obtained for PP and CB speed factors (both *r* = 0.30; CIs: 0.10–0.50 and 0.11–0.50, for PP and CB, respectively).

However, model comparison revealed that the relations with WMC could be constrained to be equal across PP and CB factors for the Search and Comparison tasks (see Appendix A). For the Substitution task, the evidence weakly supported symmetry of relations with WMC: The model comparison missed significance, while model comparison indexes AIC and BIC were inconsistent, although evidence was only weak in either direction [59].

### 3.2. Joint Analyses across Task Classes

Next, bifactor models were fitted simultaneously to all tasks administered in one administration mode (see Figure 3). Thereby, mental speed was conceptualized as a hierarchical construct with a brought general factor of mental speed and independent method components for requirements specific to Search and Comparison tasks. Cross-mode relations were allowed for the g factors of speed and for corresponding method factors. Additionally, relations with WMC were tested for all factors. The model yielded decent fit (see Appendix B).

The relations between latent variables across PP and BC mode of administration was moderately high for the g factors of speed (*r* = 0.64; CI: 0.51–0.77), corresponding with 41% shared variance. The task-specific requirements of PP and CB Search tasks were not significantly related (*r* = 0.11; CI: −0.13–0.36), whereas the specificity of the Comparison revealed a relation in the same magnitude as the g factors (*r* = 0.71; CI: 0.59–0.84). Both g factors of mental speed were moderately related with WMC, where the relation of the PP factor (*r* = 0.46; CI: 0.26–0.66) was estimated 20 points lower than that obtained for the CB factor (*r* = 0.66; CI: 0.50–0.81). None of the task-specific requirements revealed a significant relation with WMC. Model comparisons were conducted to test formally if the cross-mode correlations could be fixed to either zero or one. Analogous tests were conducted for the relations of the g factors of PP and CB speed with WMC, and if symmetry of relations with WMC could be assumed for PP and CB speed (see Appendix B). As could be expected based on the CIs of the parameter estimates, the cross-mode relation of the general speed factors could neither be fixed to zero nor one, and the same was found for the specificity of the Comparison tasks. This implies that the g factors modeled across PP and CB tasks have some variance in common, but they are clearly separable. The relation of the Search specific component could be fixed to zero without significant loss in model fit, and this more parsimonious model was preferred by the model comparison indexes AIC and BIC.

The pattern of relations with WMC was roughly comparable for factors derived from PP and from CB tasks: The moderate correlations of the g factors could neither be fixed to zero nor to one, whereas the negligible relations of the task-specific components could be removed without deterioration in model fit in both assessment modes. Critically however, model tests confirmed that the relation of CB speed with WMC was significantly higher than that estimated for PP speed with WMC. Finally, the strength of the cross-mode correlation of both speed g factors (i.e., same construct, but different methods) was compared with the relations of the respective speed factors with WMC (different construct, assessed with either the same or a different method). This confirmed that the PP g factor was less highly related with WMC than with CB speed. Conversely, the CB speed factor revealed relations of comparable strength with the PP speed factor (same construct, different method) as with WMC (different construct, same method).

One reviewer suggested to make sure that the observed only moderate PP-CB relation was not an artifact of the specified model. Hence, we tested a couple of alternative models. First, we removed WMC from the model and tested again cross-mode relations for the speed tests only. The model fit was acceptable (χ^2^ (120) = 212.81, *p* ≤ 0.01; RMSEA[CI] = 0.08 [0.06; 0.10]; SRMR = 0.11; CFI = 0.94) and cross-mode relations were virtually identical with those in the original model depicted in Figure 3 (i.e., *r* = 0.64, 0.10, and 0.71, for the g factors, the Search-specific factors, and the Search-specific factors). Next, we removed all task-specific factors, resulting in single factor models for PP and CB tasks, respectively. Removing the method factors severely deteriorated model fit (χ^2^ (134) = 663.35, *p* ≤ 0.01; RMSEA[CI] = 0.18 [0.16; 0.19]; SRMR = 0.10; CFI = 0.67), but it did not substantially change the cross-mode relation (*r* = 0.76 for the single factors). Finally, we specified full bifactor models with a general factor and task-specific factors for all task classes, either with or without WMC as a covariate. However, this resulted in severe estimation problems und inadmissible solutions.

## 4. Discussion

In the present study we tested if the traditionally purported [3,5,6] dissociation of clerical speed (Gs) and elementary cognitive speed (Gt) is artificially introduced by a method confounding related to the assessment method. To this end, we administered a battery of typical speed tasks in PP and CB mode. Hierarchical models were fitted to PP and CB speed tasks, and relations were investigated across assessment mode and with WMC as an indicator of cognitive ability.

### 4.1. Cross Mode Relations

First, separate analyses were conducted for the three types of tasks to test their equivalence across mode of administration. Even when modeling the reliable portion of variance across indicator tasks using different stimulus materials (letters, numbers, and symbols), the correlations between corresponding PP and CB task factors were of only moderate strength. Additionally, the strength of the cross mode relation depended on task type, a discrepancy that reveals the effects of task specific requirements and underscores that equivalence of different instantiations cannot be taken for granted [60].

Next, mental speed was modeled as a hierarchical construct across all tasks. However, even the broad g factors for PP and CB tasks did not share more than half of their variance. These results confirm that assessment mode strongly contributes to the dissociation of PP and CB administered speed tasks. Hence, even g factors that could be expected to be less affected by the requirements of specific tasks were shown to be clearly separable. Consequently, PP and CB tasks, respectively, appear to share an assessment-mode related communality that is also captured by their g factor and that contributes to their dissociation. The present study complements previous findings that tasks transferred from PP to CB versions (or vice versa) change their validity. This has been shown for typical Gt tasks (Sternberg, Posner, Hick, and Odd-Man-Out (OMO) tasks) that lost their validity for their original factor when transferred into PP versions, while gaining validity for the Gs factor at the same time [38,39]. Further, analogous findings have been obtained when transferring typical Gs tasks into CB versions [40]. When combining the findings obtained in the current study with the pieces of evidence obtained in earlier research, it appears that Gs and Gt are separated only by virtue of their assessment mode. Therefore, we suggest that the still widely held distinction between a clerical speed factor (Gs) and a mental speed factor (Gt) is flawed and results from an artifact of the employed assessment method.

### 4.2. Relations with WMC

The CB based speed factor revealed a higher relation with WMC than the PP based speed factor. Relation of the CB factor with WMC was of comparable strength as the cross-mode relation of both speed factors. This means that relations with a theoretically dissociable construct assessed with the same method were just as high as relations with a supposed-to-be identical construct assessed with a different method (see below for a discussion).

Additionally, the analyses conducted for separate task classes showed that the strength of this relation depends on the type of speed task. Replicating previous research [18,22,24], the relations with cognitive ability increased with complexity of the PP tasks. This rank order did not hold for the CB tasks: The Comparison tasks were of intermediate difficulty in terms of RT, but they revealed the weakest relations in relative terms. Please note, though, that empirical difficulty may serve only as a proxy for task complexity (e.g., the number of required operations).

### 4.3. Task Specificity and the Hierarchical Nature of Mental Speed

The analyses conducted separately for task types revealed different cross-mode relations, depending on task type. Analogous results were obtained with the bifactor models that decompose the variance in speed tests into a general factor of speed and factors capturing task-specific requirements (and, additionally, error). The large differences in cross-mode relations observed for the specific components were noteworthy: While the cross-mode relation for the Search specific components was virtually null, the relation of the Comparison specific components was as high as that of the g factors of speed. These large differences in (incremental) cross-mode relations arguably reflect similarity of the PP and CB implementations of the respective tasks.

These findings confirm that mental speed is best described as a hierarchical construct [39,40] and they underscore that speed tests possess specific requirements, in spite of their purported simplicity [1] (see also [61]). This implies that speed tests cannot be considered as exchangeable, and that indicators used in research and applied assessment should be carefully selected. 

The finding that none of the task specific components was related with WMC does not necessarily indicate a shortcoming of these task types. On the contrary, it suggests that task-specific requirements of these speed indicators do not have a WMC confounding. However, as a confounding method factor, they could deteriorate validity of the tasks. This can be reconciled with the finding that a higher level of aggregation across speed tasks reveals more consistent relations with intelligence than single speed tasks [38,62,63].

### 4.4. Which Factors are Responsible for the Dissociation of PP and CB Measures?

A number of factors might be responsible for the only moderate correlations of speed tests administered in PP or CB mode [30,64,65]. These can be grouped into four categories [64]: the mode of item presentation, perceptual demands, motor skill requirements, and familiarity with electronic devices, of which perceptual demands and motor skills were shown to be the most important factors [64].

In support of this, it was shown that presenting multiple stimuli at a time can distract people relative to when only one stimulus is shown [66]. Given that multiple stimuli are typically shown in PP tests while only one stimulus is typically shown in CB tests, distraction could be assumed to play a more pronounced role in PP tests as compared with CB tests. Additionally, effects of response requirements were investigated in a couple of studies. For instance, in an early study [65] response mode was substantially varied in CB tasks and resulted in a broad range of cross-mode correlations (0.28 ≤ *r* ≤ 0.61).

These differences between PP and CB tests could contribute to differential relations with cognitive ability that tend to be somewhat stronger for CB as compared with PP versions of the tests [37]. In many cases, PP tasks have considerably higher motor requirements (e.g., drawing a symbol in Substitution tasks) as compared with CB tasks where response requirements are usually minimal (e.g., pressing a button on the keyboard). Given that motor skills lack validity for higher cognitive processes, the longer time consumed for peripheral processes in PP tasks might be detrimental for their validity.

### 4.5. How to Assess Mental Speed?

The present findings clearly show that assessment mode is a decisive factor in speed test, as it contributes to the dissociation of PP and CB tasks as well as to their differential relations with cognitive ability. Both assessment modes arguably possess advantages. On the one hand, PP measures are more conventional and have been used extensively in applied assessment [8,9]. Additionally, PP tests of clerical speed served as a reference method for the broad speediness factor conceptualized in current taxonomies of mental abilities [5,6]. On the other hand, CB tests exploit the possibilities of modern technology. Administration and computation of scores can be highly automated, thereby, saving time and increasing objectivity and reliability of the measure. Additionally, richer information (e.g., item-wise RT) allows for in-depth analyses of task performance, including sophisticated modeling [31,32,33,34,35].

The finding that CB speed was found to be more highly related with WMC than PP speed with WMC is noteworthy. However, its evaluation arguably depends on theoretical perspective: From the psychometric viewpoint of clerical speed [5,6], the lower correlation of PP measures could be interpreted as reflecting better factorial or discriminant validity of the speed tests. Conversely, from the reductionist viewpoint of elementary cognitive speed [14,16], a higher correlation could be better reconciled with theoretical predictions. Reduced peripheral motor requirements [5] in the CB tasks as compared with PP tasks could contribute to this dissociation. If this was the case, it would rather speak to the use of CB measures as tests of mental speed.

Finally, it should be discussed that not all “RT tasks” qualify as appropriate measures of mental speed. Naturally, speed is conventionally scored as (inverse) RT, however, both terms should not be used interchangeably: Mental speed is a theoretical construct, whereas RT is an observable performance indicator that can be affected by a multitude of variables. It has been discussed elsewhere that most speed tests require attention control [66,67,68]. Additionally, the well-replicated finding that speed tests gain validity for cognitive ability with increasing complexity of the speed tests [18,24] suggests a confounding with the capacity of working memory. In fact, research has shown that a component capturing experimentally increasing task load in a Hick-type speed test is more clearly related with capacity than with speed of processing [69]. These findings underscore that we should not take for granted that a particular test measures mental speed only because it emphasizes fast responding or because performance is measured in a response time metric. Therefore, if one intends to measure mental speed, the selection of the measure should be clearly justified by theoretical considerations, e.g., by minimal confounding task requirements such as WMC and executive attention.

### 4.6. Limitations of the Present Study

The sample comprised mostly university students. Consequently, restrictions in ability range could have attenuated the relations observed across PP and CB measures and with WMC. However, the substantial loadings in the measurement models suggest sufficient variance in the indicators. Taken together, the only moderate cross-mode relation should be replicated in more heterogeneous samples in future research.

WMC was included as a general indicator for cognitive functioning, because it has been previously shown to be highly related with (fluid) intelligence [44,45,46,47]. However, there were neither markers of attention control that could be predicted to be related with measures of mental speed [66,67,68] nor of any of the other primary abilities. Future studies should seek completing the picture how transferring processing mode affects the pattern of relations within the nomological network of mental abilities. 

This study offered evidence that typical clerical perceptual speed tasks lose convergent validity for their original Gs factor when transferred into CB versions. Only when combining the current results with those obtained in previous studies [38,39,40], they rule likely that Gs and Gt are, in fact, separated only by virtue of their assessment mode.

## 5. Conclusions

Method factors related to the mode of assessment play a substantial role in speed tests. They contribute to the dissociation of PP and CB tests, and affect the relation of the speed measure with cognitive ability. While previous research has demonstrated that Gs and Gt tests can be transferred into the respective other task class, simply by changing their mode of administration, this study offers more direct evidence that both speed factors are different only by virtue of test mode. We suggest that the widely held distinction between clerical speed and mental speed is flawed and that unwanted issues of administration are responsible for their apparent dissociation. Both methods of measuring speed arguably have their pros and cons, but it is critical to note that they cannot be treated as exchangeable. This also holds for single measures of this multifaceted, hierarchical construct.

## Figures and Tables

**Table 1:** Descriptive statistics for paper-and-pencil and computer-based speed scores.

Speed Test	*M*	*SD*	Skew	Kurtosis
**Paper and Pencil**				
Search-Numbers	275.672	44.627	−0.129	−0.026
Search-Letters	288.248	46.068	0.027	−0.304
Search-Symbols	127.960	18.006	0.508	0.050
Comparison-Numbers	43.968	6.670	0.171	−0.335
Comparison-Letters	39.536	6.710	0.270	0.468
Comparison-Symbols	30.144	5.014	0.606	0.692
Substitution-Num→Sym	27.136	4.222	0.292	0.246
Substitution-Let→Num	29.760	4.304	0.040	−0.131
Substitution-Sym→Let	30.784	5.929	0.914	1.451
**Computer Based**				
Search-Numbers	2.783	0.259	−0.051	−0.200
Search-Letters	2.811	0.239	−0.307	0.289
Search-Symbols	2.268	0.187	−0.428	−0.216
Comparison-Numbers	1.257	0.168	−0.177	−0.455
Comparison-Letters	1.094	0.168	−0.101	−0.292
Comparison-Symbols	0.944	0.130	0.504	0.282
Substitution-Num→Sym	0.808	0.131	0.597	0.503
Substitution-Let→Num	0.867	0.149	0.476	0.518
Substitution-Sym→Let	0.917	0.121	0.281	0.486

Note. PP scores denote the number of correct responses in the given time (i.e., 45 s per test), whereas CB scores correspond with the number of correct responses per second (1 s/RT).

## Appendix A

**Table A1:** Fit of single task models.

			Difference Test						
Task/Constraint	χ^2^ (*df*)	*p*	χ^2^ (*df*)	*p*	RMSEA [CI]	SRMR	CFI	AIC	BIC
***Search***									
Unconstrained (Model 1(a); see Figure 2)	35.35 (24)	0.06	—	—	0.06 [0.00; 0.10]	0.05	0.97	1695	1754
Search_PP_ − Search_CB_ = 0	55.03 (25)	0.00	19.68 (1)	<0.001	0.10 [0.06; 0.13]	0.14	0.93	1712	1769
Search_PP_ − Search_CB_ = 1	162.44 (25)	0.00	127.08 (1)	<0.001	0.21 [0.18; 0.24]	0.13	0.67	1820	1876
Search_PP_ − WMC = Search_CB_ − WMC	37.38 (25)	0.05	2.03 (1)	0.15	0.06 [0.00; 0.10]	0.05	0.97	1695	1751
***Comparison***									
Unconstrained (Model 1(b); see Figure 2)	37.60 (24)	0.04	—	—	0.07 [0.02; 0.11]	0.05	0.98	1180	1239
Comp_PP_ − Comp_CB_ = 0	117.47 (25)	0.00	79.88 (1)	<0.001	0.17 [0.14; 0.20]	0.26	0.86	1258	1314
Comp_PP_ − Comp_CB_ = 1	118.45 (25)	0.00	80.86 (1)	<0.001	0.17 [0.14; 0.20]	0.08	0.86	1259	1315
Comp_PP_ − WMC = Comp_CB_ − WMC	37.60 (25)	0.05	<0.01 (1)	0.97	0.06 [0.00; 0.10]	0.05	0.98	1178	1234
***Substitution***									
Unconstrained (Model 1(c); see Figure 2)	30.50 (24)	0.17	—	—	0.05 [0.00; 0.09]	0.04	0.99	1174	1234
Subst_PP_ − Subst_CB_ = 0	64.81 (25)	0.00	34.31 (1)	<0.001	0.11 [0.08; 0.15]	0.19	0.93	1207	1263
Subst_PP_ − Subst_CB_ = 1	109.18 (25)	0.00	78.68 (1)	<0.001	0.16 [0.13; 0.20]	0.10	0.84	1251	1308
Subst_PP_ − WMC = Subst_CB_ − WMC	34.10 (25)	0.11	3.60 (1)	0.06	0.05 [0.00; 0.10]	0.05	0.98	1176	1233

Note. PP = paper-and-pencil tasks, BC = computer-based tasks. Difference Test = change in χ^2^ for the constrained models relative to their unconstrained parent model. CI = 95% confidence interval.

## Appendix B

**Table A2:** Fit of Bifactor Models.

	Difference Test								
Model/Constraint	χ^2^ (*df*)	*p*	χ^2^ (*df*)	*p*	RMSEA [CI]	SRMR	CFI	AIC	BIC
Unconstrained (Model 2; see Figure 3)	272.90 (168)	0.00	—	—	0.07 [0.06; 0.09]	0.10	0.94	1896	2074
***Relations PP* − *CB***									
Speed_PP_ − Speed_CB_ = 0	320.48 (169)	0.00	47.59 (1)	<0.001	0.08 [0.07; 0.10]	0.22	0.91	1941	2117
Speed_PP_ − Speed_CB_ = 1	384.66 (169)	0.00	111.76 (1)	<0.001	0.10 [0.09; 0.11]	0.11	0.87	2005	2181
Search_PP_ − Search_CB_ = 0	273.66 (169)	0.00	0.76 (1)	0.38	0.07 [0.05; 0.09]	0.10	0.94	1894	2070
Comp_PP_ − Comp_CB_ = 0	327.89 (169)	0.00	54.99 (1)	<0.001	0.09 [0.07; 0.10]	0.12	0.91	1949	2124
Comp_PP_ − Comp_CB_ = 1	299.67 (169)	0.00	26.77 (1)	<0.001	0.08 [0.06; 0.09]	0.09	0.92	1920	2096
***Relations PP* − *WMC***									
Speed_PP_ − WMC = 0	288.21 (169)	0.00	15.31 (1)	<0.001	0.08 [0.06; 0.09]	0.14	0.93	1909	2084
Speed_PP_ − WMC = 1	308.05 (169)	0.00	35.15 (1)	<0.001	0.08 [0.07; 0.10]	0.10	0.92	1929	2104
Search_PP_ − WMC = 0	274.86 (169)	0.00	1.96 (1)	0.16	0.07 [0.06; 0.09]	0.10	0.94	1896	2071
Comp_PP_ − WMC = 0	273.00 (169)	0.00	0.10 (1)	0.75	0.07 [0.05; 0.09]	0.10	0.94	1894	2069
***Relations CB* − *WMC***									
Speed_CB_ − WMC = 0	310.27 (169)	0.00	37.37 (1)	<0.001	0.08 [0.07; 0.10]	0.14	0.92	1931	2106
Speed_CB_ − WMC = 1	296.45 (169)	0.00	23.51 (1)	<0.001	0.08 [0.06; 0.09]	0.10	0.92	1917	2093
Search_CB_ − WMC = 0	273.03 (169)	0.00	0.13 (1)	0.71	0.07 [0.05; 0.09]	0.10	0.94	1894	2069
Comp_CB_ − WMC = 0	273.03 (169)	0.00	0.14 (1)	0.71	0.07 [0.05; 0.09]	0.10	0.94	1894	2069
***Testing the Symmetry of Relations***									
Speed_PP_ − WMC = Speed_CB_ − WMC	277.40 (169)	0.00	4.50 (1)	0.03	0.07 [0.06; 0.09]	0.10	0.94	1898	2073
Speed_PP_ − Speed_CB_ = Speed_PP_ − WMC	276.58 (169)	0.00	3.68 (1)	0.06	0.07 [0.06; 0.09]	0.11	0.94	1897	2073
Speed_PP_ − Speed_CB_ = Speed_CB_ − WMC	272.92 (169)	0.00	0.02 (1)	0.88	0.07 [0.05; 0.09]	0.10	0.94	1894	2069

Note. PP = paper-and-pencil tasks, BC = computer-based tasks. Difference Test = change in χ^2^ for the constrained models relative to their unconstrained parent model. CI = 95% confidence interval.
